# Comparison of antioxidant capacities and antioxidant components of commercial bitter melon (Momordica charantia L.) products

**DOI:** 10.3906/kim-2007-67

**Published:** 2020-12-16

**Authors:** Esin AKYÜZ, Sercan TÜRKOĞLU, Kevser SÖZGEN BAŞKAN, Esma TÜTEM, Mustafa Reşat APAK

**Affiliations:** 1 Department of Chemistry, Faculty of Engineering, İstanbul University-Cerrahpaşa, İstanbul Turkey; 2 Sem Laboratory Equipments Marketing Industry and Trade Inc., İstanbul Turkey

**Keywords:** *Momordica charantia*, bitter melon, total antioxidant capacity, total phenolic content, CUPRAC, ABTS, HPLC

## Abstract

In this study, the total phenolic contents and total antioxidant capacities of some commercial bitter melon products (powder, packaged powder, capsule, paste in olive oil), and of unripe and ripe fruits were determined by spectrophotometric and chromatographic methods. The total antioxidant capacities of unripe and ripe bitter melon samples, determined by using the CUPRAC (cupric reducing antioxidant capacity assay) and ABTS (2,2′-azino-bis(3-ethylbenzthiazolin-6-sulfonic acid))/HRP (horseradish peroxidase) methods, were 42.5 and 36.3 µmol TRE (Trolox equivalent) g–1, and 8.7 and 7.0 µmol TRE g–1, respectively. The TAC (total antioxidant capacity) order of the studied samples using the same 2 methods were determined as follows: capsule (CUPRAC value, 140.8; ABTS/HRP value, 143.6 µmol TRE g–1) > packaged powder (129.6; 126.1) > powder (52.3; 64.3) > unripe fruit (42.5; 36.3) > paste in olive oil (17.6; 14.4) > ripe fruit (8.7; 7.0). The order of phenolic content was found as follows: unripe fruit (193.2 µmol GAE (gallic acid equivalent) g-1) > capsule (162.0) > packaged powder (160.6) > powder (83.6) > paste in olive oil (38.3) > ripe fruit (14.6).

## 1. Introduction

Bitter melon, a member of the *Cucurbitaceae* family, is a tropical plant with the Latin name *Momordica charantia* . It is also known by different names such as bitter gourd, African cucumber, balsam apple, balsam pear, balsam pear, papillae, and karela [1].

Bitter melon, originating from India, likes warm, humid regions and grows naturally in Asia, Africa, USA, Australia, Brazil, China, Iran, Malaysia, Thailand, and Turkey [2–4]. It is cultivated in South America and Far Eastern countries for both treatment of certain diseases and food use. Bitter melon in Turkey is grown from seed in May mostly in Yalova, the environs of Bursa, and the Aegean region [5]; ripe fruits are harvested in August.

The plant is composed of 83.2% moisture, 2.9% protein, 1% fat, 9.8% carbon, 1.7% fiber, and 1.4% mineral and organic substances (calcium, phosphorus, iron, carotene, thiamine, nicotinic acid, riboflavin, ascorbic acid, copper, and potassium) [6]. Bitter melon contains high amounts of vitamin C, as well as vitamin A, β-carotene, α-carotene, potassium, magnesium, and zinc. The membranes of the seeds of the ripe fruits are a good source of lycopene [7–9]. Leaves and unripe fruits of the plant, rich in vitamins and minerals, are consumed as vegetables. Since the taste of the ripe fruits is bitter, the unripe form is preferred when consumed as a vegetable [10–11]. Unripe fruits, ripe fruits, preparations made from whole plants, and teas prepared from leaves are used for treatment or health protection.

It has been shown that extracts obtained from the fruit have scavenging activity for free radicals, superoxide and hydroxyl radicals that cause pathophysiological changes [12]. In addition to its antitumor, antibacterial, antiviral, antigenotoxic, antioxidant, antihepatoxic, antipyretic, and degassing effects, bitter melon is an important plant used in alternative treatments for diseases such as rheumatoid arthritis, gout, intestine, leprosy, jaundice, anemia, malaria, hypertension, and low cholesterol. Detoxification of the body, maintaining a certain balance of hormones in the brain, improving immunity, increasing luteinizing hormone, and preventing different tumors are the other reported benefits of this plant [5,13–17].

Commercial food supplements, which are prepared from various fruits, vegetables, and antioxidant rich plants, have recently attracted much attention. Food supplements consist of vitamins, minerals, amino acids, fatty acids, and other substances delivered in the form of pills, tablets, capsules, powder, extract, tea, etc. People use these products, which are believed to play an important role in preventing diseases, for a healthy life and well-being. Nearly two-thirds of the world’s population still prefers local folk medicine to industrial medicines, in part for economic reasons, but in most cases due to a traditional lifestyle. Interest in herbal medicinal products is also increasing in Western countries because such drugs are considered “more natural” and therefore less toxic (although this is not always true) [18,19]. Because food supplements are formulated from different plant species and have different compositions and concentrations of active ingredients, they may be expected to show wide variability in antioxidant power.

Bitter melon extract (due to its bitterness) is incorporated in the form of supplements that can be consumed by individuals to get the benefits of its medicinal properties. The extraction process of bitter melon is carried out through 2 different methods. The active components of the fruit are acquired in the bitter melon extract through the extraction processes. Bitter melon has a wide array of vitamins and minerals that are essential for humans. Along with these nutrients, bitter melon also has antioxidant properties that make the extract very beneficial. The extract is commercially available only in the form of a powder used to manufacture products that are readily available to consumers [20]. Bitter melon extracts and other related commercial products are also sold in Turkey.

In all studies related to bitter melon published to date, the antioxidant activities of the whole fruit or parts such as leaves, peels, and seeds have been examined, but no research has been done on commercial products prepared from this plant. In addition, a comparison of the antioxidant activity of unripe and ripe fruits of bitter melon and their different commercial products has not been made. The objective of this study is to compare unripe and ripe bitter melon fruits supplied from producers in Turkey and some commercial bitter melon products bought from local spice shops in terms of total phenolic content and total antioxidant capacities. The CUPRAC method [21], which is widely used in antioxidant capacity determinations, was used for the first time in this study for the antioxidant capacity measurements of the mentioned samples. This reagent is much more stable and easily accessible than the chromogenic radical reagents (e.g., ABTS, DPPH, etc.) The TAC values of antioxidants found with CUPRAC are perfectly additive, i.e. the TAC of a phenolic mixture is equal to the sum of TAC values of its constituent polyphenols [21,22]. Combined high performance liquid chromatography (HPLC)–CUPRAC [23–25] was used to determine the components contributing to antioxidant capacity. This method gives a reliable estimate of the actual antioxidant capacity of sample extracts. The TAC values found by the combined HPLC–CUPRAC method were compared with those of the spectrophotometric CUPRAC method.

## 2. Materials and methods

### 2.1. Reagents and instrumentation

Neocuproin (Nc) (2,9-dimethyl-1,10-phenanthroline), copper(II) chloride dihydrate, (-)-epicatechin, gallic acid monohydrate, (+)-catechin hydrate, chlorogenic acid, rutin (quercetin-3- *O* -rutinosid) hydrate, *p* -coumaric acid, ethanol (EtOH), acetonitrile, methanol (MeOH), acetone, acetic acid, horseradish peroxidase (HRP), ABTS (2,2′-azino-bis(3-ethylbenzthiazolin-6-sulfonic acid)), hydrogen peroxide (H2O2), and FC (Folin–Ciocalteu) reagent were supplied from Sigma (Steinheim, Germany); trolox and quercetin from Sigma-Aldrich Chemie GmbH (Steinheim, Germany); vanillic acid and copper (II) sulfate pentahydrate from Fluka Chemie AG (Buchs, Switzerland); benzoic acid, potassium dihydrogen phosphate and hydrochloric acid (HCl) from Merck KGaA (Darmstadt, Germany); ammonium acetate (NH4Ac), sodium hydroxide (NaOH), sodium carbonate, and disodium hydrogen phosphate from Honeywell Riedel-de Haën GmbH (Seelze, Germany). All chemicals used were of analytical reagent grade.

The instruments and equipment used were as follows: Shimadzu brand ATX224 analytical balance for weighing chemicals and real samples (Shimadzu Corp., Kyoto, Japan), Bandelin Sonorex model ultrasonic bath for preparation of solutions and extracts (Bandelin electronic GmbH & Co. KG, Berlin, Germany), HI 221 Calibration Check Microprocessor pH-meter for determining pH values of solutions (Hanna Instruments, Inc., Smithfield, RI, USA), Varian Cary 100 UV-visible spectrophotometer for absorbance measurements (Varian, Inc., Palo Alto, CA, USA), Waters 1525 HPLC with photodiode array (PDA) detector for HPLC analysis (Waters Corp., Milford, MA, USA), WiseVen WOV brand vacuum oven (Witeg Labortechnik GmbH, Wertheim, Germany), WiseCube brand WIS-20 model shaker (Witeg Labortechnik GmbH), IKA HB4 Basic brand water bath (IKA-Werke Gmbh & Co. KG, Staufen, Germany), Select brand vortex, Millipore brand bidistilled water device (EMD Millipore Corp., Burlington, MA, USA), IKA RV05 Basic brand evaporator, and Telstar brand freeze-dryer for drying natural samples (Telstar North America Inc., Bristol, PA, USA).

### 2.2. Preparation of solutions

The stock solutions of gallic acid, gentisic acid, catechin, vanillic acid, chlorogenic acid, syringic acid, epicatechin, *p* -coumaric acid, benzoic acid, rutin, and quercetin were prepared in 80% (v/v) MeOH–water. All antioxidant solutions were stored at −20 °C.

The spectrophotometric assay reagents were prepared as follows. CUPRAC method: 1.0 × 10–2 M Cu(II) chloride solution and 1 M NH4Ac buffer (pH 7.0) solution were prepared in distilled water, 7.5 × 10–3 M Nc solution in 96% ethanol.

ABTS/HRP method: 4.0 × 10–3 M ABTS solution, 1.2 × 10–4 M hydrogen peroxide solution, 2.4 × 10–5 M HRP solution were prepared in phosphate buffer pH 7.4.

FC method: for Lowry A solution, 0.1 M NaOH solution was prepared in distilled water, 2% (w/v) Na2CO3 solution was prepared in 0.1 M NaOH solution. For Lowry B solution, 1% (w/v) NaKC4H4O6 solution was prepared in distilled water, 0.5% (w/v) CuSO4 solution was prepared in 1% NaKC4H4O6 solution. For Lowry C solution, 1 mL of Lowry B solution was added to 50 mL of Lowry A solution.

### 2.3. Preparation of bitter melon samples for analysis

#### 2.3.1. Supply of bitter melon samples

Powder, paste in olive oil, capsules, and packaged powder bitter melon samples were obtained from local herbal product sellers. Unripe and ripe bitter melon fruits were directly supplied from a grower who raised this plant in Silifke (Mersin, Turkey).

#### 2.3.2. Preparation of bitter melon samples

Powder, packaged powder, and capsule bitter melon samples were weighed directly because they were already dry and powdered. The paste in olive oil was filtered with a cheesecloth to separate it from the fatty portion as it was a fatty pulp; the obtained pulp was dried on filter paper under laboratory conditions. The unripe and ripe bitter melon fruits, whose seeds were removed, were cut into thin pieces with a plastic knife and used after freeze-drying at −40 °C.

#### 2.3.3. Extraction of bitter melon samples

To determine the most suitable extraction solvent, extraction was carried out using MeOH, EtOH solvents, and their aqueous solutions [the volume percentage of these solvents were 80% and 50% (v/v)]. Dried bitter melon samples were extracted with one of these solvents. For this purpose, 2 g amounts of the dry samples were weighed and extracted in 3 steps in stoppered flasks in an ultrasonic bath for 15 min with 20 mL of solvent. The upper phase was decanted and extracted for a second time with another 20 mL of solvent. Final extraction was performed for a third time with another 10 mL for 15 min. These 3 extracts were combined to make the final volume up to 50 mL. The extracts were then passed through a GF/PET (glass fiber/polyethylene terephthalate) 1.0/0.45-µm microfilter and stored at −20 °C before analysis.

### 2.4. Spectrophotometric methods

#### 2.4.1. CUPRAC method

One mL each of 1.0 × 10–2 M copper (II) chloride, 7.5 × 10–3 M Nc, and 1 M NH4Ac buffer (pH 7.0) were added to a test tube. After adding (x) mL of antioxidant or sample solution to this mixture, the volume was completed to 4 mL with distilled water. The stirred solutions were allowed to stand at room temperature for 30 min. At the end of this period, the absorbance of the prepared mixtures was measured at 450 nm against the reference solution [21].

The total antioxidant capacities of the bitter melon samples were calculated as µmol TRE g–1 dry sample by using the calibration line formed between absorbance and concentration of trolox (TR) standard. For the CUPRAC method: eTR = 15000 L mol–1 cm–1 (in 80% MeOH).

#### 2.4.2. ABTS/HRP method

This method, originally described by Arnao et al. [26], was partially modified in regard to reagent amounts and duration. One milliliter each of 4.0 × 10–3 M ABTS, 2.4 × 10–5 M HRP and 1.2 × 10–4 M H2O2 solutions were added to a test tube. The mixture was allowed to stand for 5 min to stabilize the absorbance of the formed ABTS radical (ABTS •+). At the end of this period, (x) mL of the sample was added and the total volume was completed to 4 mL with distilled water and left to stand for another 5 min. Absorbances were recorded at 10th min against pH 7.4 phosphate buffer at 730 nm. The absorbance difference (ΔA) was calculated by subtracting the absorbance of the sample solution from the absorbance of the blank radical solution (containing solvent instead of sample).

The total antioxidant capacities of the bitter melon samples were calculated as µmol TRE g–1 dry sample by using the calibration line formed between absorbance and concentration of TR standard. For the ABTS/HRP method: εTR = 22000 L mol–1 cm–1 (in 80% MeOH).

#### 2.4.3. FC method

According to the FC method measuring total phenolic content (TPC), (x) mL sample solution, (2-x) mL distilled water, and 2.5 mL of Lowry C solution (formed by the addition of 50 mL Lowry A solution to 1 mL Lowry B solution) were added to a test tube. After 10 min, 0.25 mL FC reagent, diluted with water at a 1:3 (v/v) ratio, was added. The tubes were kept at room temperature for 30 min and their absorbances were measured at 750 nm against a reference solution [27].

The TPC of bitter melon samples was calculated as µmol GAE g–1 dry sample using the calibration line formed between the absorbance and the concentration of GA (gallic acid) standard. For the FC method: εGA = 6100 L mol–1 cm–1 (in 80% MeOH).

### 2.5. Chromatographic method

For chromatographic analysis of the polyphenolic compounds found in bitter melon samples, a gradient elution program was developed using a Supelco C18 (5 µm, 25 cm × 4.6 mm) column (Supelco, Inc., Bellefonte, PA, USA) and bidistilled water containing 3% (v/v) acetic acid (A) and MeOH (B) solvents. A total time of 60 min, including washing and balancing, was allowed for the gradient elution program, which was applied as follows: initially 100% A, 2 min from 100% to 99% A (curve 10), 2 min from 99% to 97% A (curve 10), 4 min from 97% to 95% A (curve 6), 2 min from 95% to 90% A (curve 6), 10 min from 90% to 75% A, 10 min from 75% to 65% A (curve 6), 10 min from 65% to 50% A (curve 6), 10 min from 50% to 30% A (curve 6), 10 min from 30% to 100% A (curve 6). Curve numbers in parentheses are the slope (change rate of solvent) codes of the Empower Software (Waters Corporation) program. The flow rate was 1 mL min–1; analytical detection wavelengths were selected as 270 nm to observe all phenolic constituents.

In the HPLC analysis of bitter melon extracts, retention times and PDA detector spectra were compared with those of standards. Peak areas were used for quantitative analysis of preidentified polyphenolic compounds.

### 2.6. Combined HPLC–CUPRAC method

The contribution of the components of unripe and ripe bitter melon samples and commercial products to the observed total antioxidant capacity was calculated using Equation (1) [23–25]. The component concentrations determined by HPLC were multiplied by TEAC (Trolox equivalent antioxidant capacity) coefficients determined by the spectrophotometric method, and total antioxidant capacities of bitter melon samples were determined. HPLC–CUPRAC refers to the capacity calculated by multiplying the concentrations determined in HPLC by the TEAC coefficients pertaining to the CUPRAC method. Since these calculated capacity values were based on both chromatographic and spectrophotometric data, the symbols of both instrumental techniques were used to express them.

(1)

Ci: concentration of ith component determined by HPLC; TEACi: TEAC coefficient of ith component calculated by the selected TAC measurement method (i.e. CUPRAC).

### 2.7. Statistical analysis

Spectrophotometric assays were applied in triplicate for each sample and standard. Descriptive statistical analyses were performed using Excel software (Microsoft Office 2016) for calculating the mean and the standard error of the mean (these values are not presented as a table). In addition, the Pearson correlation coefficient was calculated using Excel to compare the TAC values found with the CUPRAC and ABTS methods.

## 3. Results and discussion

### 3.1. Preparation of bitter melon samples and selection of the extraction solvent

The unripe and ripe bitter melon fruits were dried with a freeze-dryer and ground using a mortar. As described in the study of Horax et al. [28], the bitter melon varieties were prepared for analysis by drying with 2 different methods (oven and freeze-dryer), and thus the effect of the drying method was examined. The TPC of the studied samples were determined as 5.39‒8.94 mg chlorogenic acid equivalent (CAE) per gram for oven-dried samples and 4.64‒8.90 mg CAE g–1 for freeze-dried samples, showing that the different drying methods did not cause a significant difference in the TPC of samples. Therefore, we preferred to dry our samples with the freeze-dryer.

**Figure 1:**
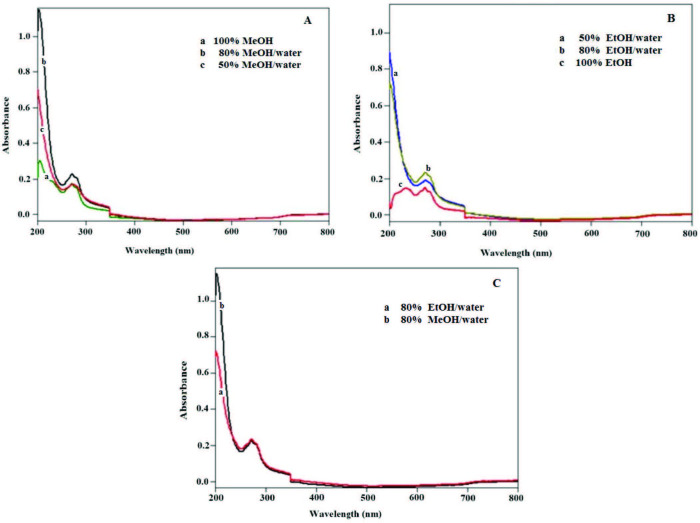
Spectra of extracts of powdered bitter melon sample containing MeOH-water (A) and EtOH-water (B) in different proportions (100%, 80%, 50%), and the comparison of the spectra of 80% MeOH-water and 80% EtOH-water (C) extracts.

Figure 1A shows the UV-visible spectra of ultrasonic bath extracts prepared at 50% (v/v) MeOH–water, 80% (v/v) MeOH–water, and 100% MeOH solvent ratios of the powder bitter melon sample. Figure 1B shows the UV-visible spectra of extracts prepared in EtOH solvent at the same alcohol/water ratios. The comparison of the spectra of 80% MeOH–water and 80% EtOH–water extracts is shown in Figure 1C, showing no significant difference between the extraction efficiencies of these 80% alcohol solutions. The spectral peak appearing around 280 nm (Figures 1A–1C) is conventionally adopted for phenol detection in HPLC analysis of phenolic substances recovered from various sources [29]. In the literature, since MeOH has a protective role inhibiting oxidation of phenolic compounds with enzymes such as phenoloxidase, 80% (v/v) MeOH was chosen as the most suitable extraction solvent [30]. In most of the research studies on bitter melon, it was stated that EtOH, MeOH, or water was suitable as extraction medium [31–33].

### 3.2. Spectrophotometric analysis of bitter melon samples

#### 3.2.1. Total antioxidant capacities and total phenolic contents

**Figure 2:**
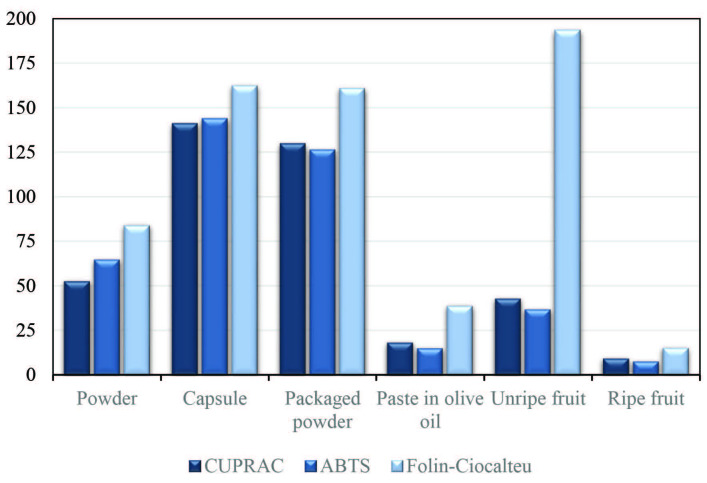
Comparison of CUPRAC (μmol TRE g–1), ABTS/HRP (μmol TRE g–1), and Folin (μmol GAE g–1), method findings of bitter melon samples.

The TAC values of bitter melon samples were determined by CUPRAC and ABTS/HRP methods, and the TPC values by the FC method. The findings of all tested bitter melon samples were compared with the bar diagram (Figure 2).

The TAC order of the studied samples was the same with respect to the findings of both CUPRAC and ABTS/HRP methods, and was capsule > packaged powder > powder > unripe fruit > paste in olive oil > ripe fruit. Sorting in terms of phenolic components yielded unripe fruit > capsule > packaged powder > powder > paste in olive oil > ripe fruit (Figure 2). The Pearson correlation coefficient (r = 0.9936, P-value = 0.0001) showed that the TAC values measured by the CUPRAC method were strongly positively correlated with those of the ABTS method.

The total phenol content of the unripe fruit was found to be quite high compared to the ripe fruit and other samples. On the other hand, despite its high TPC, the value of TAC was low. This may correspond to the presence of reducing components that respond to the FC method but do not have antioxidant properties [9]. This should be taken as a natural consequence of the indefinite but presumably high redox potential of FC reagent, which can easily oxidize certain weak reductants (besides polyphenols) other than antioxidants [27]. In a study by Kubola and Siriamornpun [34], TPC values (as mg GAE g-–1) of methanolic extracts of dry bitter melon samples were determined as 324 ± 1.63 for green fruits and 224 ± 0.86 for ripe fruits. In the same study, it was stated that TPC findings were compatible with the findings of the FRAP (ferric reducing antioxidant power) method used in the determination of TAC (R2 = 0.948). The TAC values (as µmol FeSO4 g–1) determined by the FRAP method were 43.8 ± 0.008 for green fruit and 9.41 ± 0.007 for ripe fruit. These values are compatible with our findings.

It is known that bitter melon contains a variety of antioxidant compounds such as water-soluble vitamin C, lipophilic vitamin E, and carotenoids (carotene, xanthophylls, and zeaxanthin) [35]. The higher TAC value of unripe bitter melon fruit can be associated with their high vitamin C content. As the fruit ripens, the vitamin C content decreases and the carotenoid content increases (orange color formation). In the study by Goo et al. [36], the change in vitamin C content was observed to depend on the ripening stage of the fruit. After the fruit has formed, samples were collected every 5 days between 5–20 days and vitamin C contents were determined. The vitamin C contents of samples were 2093, 2344, 1938, and 1767 mg/100 g, respectively. Vitamin C was found to be highest in the fruit collected after 10 days. In our study, the TAC value of the paste in olive oil was found to be lower than those of other samples. This can be explained by the fact that lipophilic compounds (especially carotenoids) have passed into the oil while the ripe fruit is kept in oil.

In the literature, the primary methods used to determine the antioxidant activity of bitter melon fruit were DPPH (2,2-diphenyl-1-picrylhydrazyl), ABTS, FRAP, linoleic acid methyl ester oxidation [28,31,33,37–39], Fe (III) thiocyanate, thiobarbituric acid [32], and b-carotene bleaching [38] assays. Some disadvantages of the DPPH and FRAP methods, which are widely used in the measurement of antioxidant activity of polyphenolic compounds, have been mentioned in the literature. The DPPH method does not provide a competitive reaction because DPPH color can be lost via a radical reaction (hydrogen atom transfer) or chemical reduction (single-electron transfer) as well as through unrelated reactions, and steric inaccessibility of the reagent is a major determinant of the reaction. Thus, small molecules that have better access to the radical site exhibit a higher apparent antioxidant capacity with this test. Furthermore, DPPH has been criticized because it is a stable nitrogen radical that bears no similarity to the highly reactive and transient peroxyl radicals actually involved in lipid peroxidation. Many antioxidants that react quickly with peroxyl radicals may react slowly or may even be inert to DPPH due to steric inaccessibility [39]. Ou et al. [40] stated that the FRAP method is unable to detect slowly reacting polyphenolic compounds. Similarly, Blasa et al. [41] reported on the FRAP method’s limitations; when FRAP was used to determine the antioxidant potential of polyphenols in water and methanol (i.e. the solvent typically used for extraction of antioxidants), the change in absorbance continued after 4 min. Therefore, the FRAP values for these compounds cannot be accurately determined within 4 min. In another study in which similar comments were made for both methods mentioned, it was stated that the kinetics of the CUPRAC method were faster [42]. There is only one study using the CUPRAC method for the determination of unripe and ripe bitter melon fruits, but the results were compared to the absorbance of the butylated hydroxy toluene (BHT) standard [43]. In addition, a comparison of the phenolic content and antioxidant capacity of bitter melon fruits and commercial products does not exist in the literature.

### 3.3. Chromatographic analysis results

#### 3.3.1. Application of HPLC method to various polyphenolic antioxidant standards

**Figure 3:**
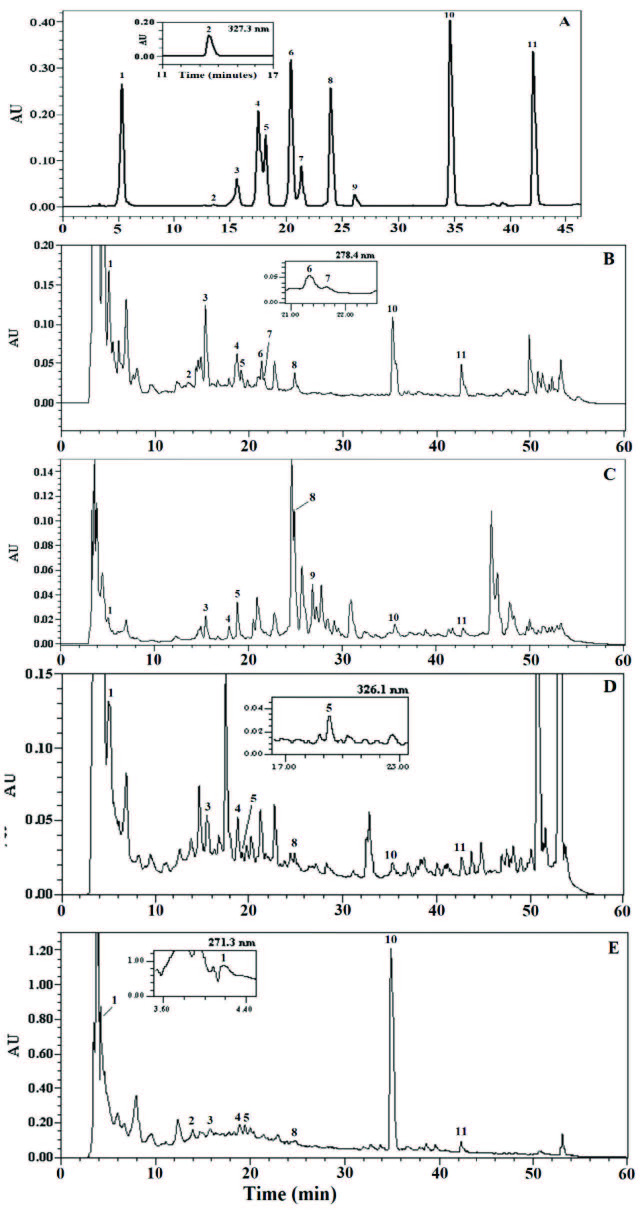
Chromatograms (at 270 nm) of the synthetic mixture containing various antioxidant standards (A), 80% MeOH (v/v) extracts of unripe fruit (B), ripe fruit (C), powder (D), and capsule products (1: Gallic acid, 2: Gentisic acid, 3: Catechin, 4: Vanilic acid, 5: Chlorogenic acid, 6: Syringic acid, 7: Epicatechin, 8: p-Coumaric acid, 9: Benzoic acid, 10: Rutin, 11: Quercetin).

In the studied bitter melon samples, the chromatogram of the standards of the phenolic compounds determined by the developed HPLC method is shown in Figure 3A. The data obtained from the calibration graphs created between the concentrations of the standards and peak areas are shown in Table 1.

**Table 1:** The HPLC method findings of standard phenolic compounds.

Phenolic compound	Calibration equation	Correlationcoefficient (r)	Linear range (mol L−1)
Gallic acid	y = 1.32 × 1010 c – 4.41 × 105	0.9999	8 × 10–5 – 8 × 10–4
Gentisic acid	y = 0.59 × 1010 c – 1.69 × 105	0.9998	8 × 10–5 – 8 × 10–4
Catechin	y = 0.44 × 1010 c – 1.60 × 105	0.9998	8 × 10–5 – 8 × 10–4
Vanilic acid	y = 1.54 × 1010 c – 4.47 × 105	0.9993	8 × 10–5 – 8 × 10–4
Chlorogenic acid	y = 2.60 × 1010 c – 8.39 × 105	0.9999	8 × 10–5 – 8 × 10–4
Syringic acid	y = 1.51 × 1010 c – 3.45 × 105	0.9995	8 × 10–5 – 8 × 10–4
Epicatechin	y = 0.49 × 1010 c – 1.02 × 105	0.9996	8 × 10–5 – 8 × 10–4
p-Coumaric acid	y = 3.00 × 1010 c – 7.39 × 105	0.9995	8 × 10–5 – 8 × 10–4
Benzoic acid	y = 0.15 × 1010 c – 0.65 × 105	0.9990	8 × 10–5 – 8 × 10–4
Rutin	y = 2.78 × 1010 c – 7.40 × 105	0.9998	8 × 10–5 – 8 × 10–4
Quercetin	y = 2.67 × 1010 c – 7.53 × 105	0.9996	8 × 10–5 – 8 × 10–4

In the calibration equations given in Table 1, the symbols used are y: peak area, c: concentration, and r: correlation coefficient.

#### 3.3.2. HPLC analysis of bitter melon samples with HPLC–CUPRAC calculation of TAC

The studied bitter melon sample chromatograms (at 270 nm) are shown in Figures 3B–3E. The concentrations of the compounds determined from chromatograms were calculated with the help of the calibration equations given in Table 1; theoretical TAC calculation was performed using the HPLC–CUPRAC method (Table 2). The spectrophotometric TAC values of the studied samples were higher than those obtained by the HPLC–CUPRAC method primarily because chromatographic calculations are based on precisely identified compounds. There are unidentified peaks in the chromatograms because they do not have standards. Antioxidant compounds identified in almost all test samples were gallic acid, gentisic acid, catechin, vanillic acid, chlorogenic acid, syringic acid, *p* -coumaric acid, rutin (quercetin-3- *O* -rutinosid), and quercetin. Unlike other studies in the literature, rutin and quercetin were also determined in the samples we examined.

**Table 2:** The antioxidant capacity values of phenolic compounds found in bitter melon samples determined by HPLC-CUPRAC method (µmol TRE g–1).

Phenolic compounds	Powder	Capsule	Packagedpowder	Paste inolive oil	Unripe fruit	Ripe fruit
Gallic acid	2.1	-	1.9	-	5.1	0.4
Gentisic acid	-	4.0	3.9	-	1.3	-
Catechin	2.2	5.2	-	-	8.4	0.9
Vanilic acid	0.3	0.9	0.7	0.3	1.5	0.1
Chlorogenic acid	0.3	1.4	1.5	0.5	1.0	0.3
Syringic acid	-	-	-	0.2	0.4	-
Epicatechin	-	-	-	-	-	-
p-Coumaric acid	0.1	-	-	0.1	0.2	0.1
Rutin	0.3	6.3	4.6	1.2	1.9	0.2
Quercetin	0.6	1.2	0.9	0.8	1.7	0.5
TAC	5.9 (11.3%)a	19.0(13.5%)	13.5(10.4%)	3.1(7.6%)	21.5(55.3%)	2.5(28.7%)

aThe values given in parentheses are % of the ratio of TAC value determined by the HPLC-CUPRAC method to TP value determined by the CUPRAC method.

It has been stated by many researchers that bitter melon contains phenolic compounds with antioxidant properties [9,28,33,34,37,44]. It has also been reported that the amounts of these compounds differ depending on the type of fruit, harvest time, environmental factors (temperature, light, etc.), and growing conditions (greenhouse, field, fertilizer used, etc.). In different studies, phenolic compounds determined in bitter melon fruit were reported to be gallic acid, gentisic acid, catechin, epicatechin, caffeic acid, and chlorogenic acid. For example, the amount of gallic acid determined in the fleshy portions of 4 types of freeze-dried bitter melon samples was in the range of 8.04–13.74 mg 100 g–1 [9]. In another study, the gentisic acid content of oven-dried bitter melon samples was given as 0.9–5.91 mg g–1. In dried bitter melon fruit, catechin determination was performed using subcritical water extraction (SWE), methanol extraction and Soxhlet water extraction techniques; the catechin amounts were 46.16, 1.61, and 1.77 mg g–1, respectively [44]. Kubola and Siriamornpun [34] determined the caffeic acid concentration as 3.55 mg L–1 in the methanol extract of bitter melon. The chlorogenic acid concentrations were determined in a study in which 4 kinds of bitter melon samples were examined; they were reported as 6.42–14.15 mg 100 g–1 in freeze-dried samples and 4.55–16.37 mg 100 g–1 in oven-dried samples [9].

The most important difference of our study from other studies on this subject is that the ratio of the theoretical TAC value (as µmol TRE g–1) calculated using the HPLC–CUPRAC equation to the spectrophotometric TAC value (as µmol TRE g–1) determined by the CUPRAC method has been reported. In fact, the individual contribution of each compound determined by HPLC to the overall TAC was also calculated. The highest percentages were determined for the unripe and ripe fruits as 55.3% and 28.7%, respectively (Table 2).

## 4. Conclusion

Studies on efficient sources of natural antioxidants to replace synthetic antioxidants added to foods as preservatives continue to attract current interest. In this regard, bitter melon ( *Momordica charantia* L.) is one of the important herbal products grown in Turkey, used widely as food or for the treatment of certain diseases. Our study is the first to identify and quantify the bioactive compounds (i.e. antioxidant compounds and phenolics) of the locally produced bitter melon and its commercial products. Our findings showed that the essential bioactive components unique to bitter melon (especially phenolic acids and flavonoids known to be antioxidants) are found in higher amounts in commercial products. This finding was to be expected, because although there is no information on the labels of these products regarding their preparation procedure, the capsule and powder forms of the plants are known to contain high amounts of phytochemicals [45]. In addition, the phenolic content of the unripe fruit was found to be higher than that of the ripe fruit, which is again compatible with literature results.

It is emphasized in most research that the most common problems observed in dietary supplements are the risk of contamination, additives, toxicity, standardization of dose, and accuracy of labeling. For this reason, standardization in analytical procedures, i.e. efficient use of high precision analytical methods to analyze these products, is important for the protection and safety of consumers and efficient evaluation of plant resources. For accuracy and reliability, the analysis must be performed by well-trained people in authorized laboratories. As a result, standard analytical methods (i.e. spectrophototometric methods combined with HPLC) used in this work to determine the TAC and TPC of this plant can be suggested for analyzing similar plants and their commercial products.
